# Retinal vascular reactivity in carriers of X-linked inherited retinal disease – a study using optical coherence tomography angiography

**DOI:** 10.3389/fopht.2024.1415393

**Published:** 2024-07-09

**Authors:** Sena Ayse Gocuk, Xavier Hadoux, Charmaine Catipon, Elise Cichello, Himeesh Kumar, Jasleen Kaur Jolly, Peter van Wijngaarden, Thomas Llewelyn Edwards, Lauren Nicole Ayton, David Cordeiro Sousa

**Affiliations:** ^1^ Department of Optometry and Vision Sciences, The University of Melbourne, Melbourne, VIC, Australia; ^2^ Centre for Eye Research Australia, Royal Victorian Eye and Ear Hospital, Melbourne, VIC, Australia; ^3^ Ophthalmology, Department of Surgery, The University of Melbourne, Melbourne, VIC, Australia; ^4^ Vision and Eye Research Institute, Anglia Ruskin University, Cambridge, United Kingdom

**Keywords:** carrier, females, X-linked, inherited retinal disease, OCT-A, retinal vasculature

## Abstract

**Purpose:**

Female carriers of X-linked inherited retinal diseases (IRDs) can show highly variable phenotypes and disease progression. Vascular reactivity, a potential disease biomarker, has not been investigated in female IRD carriers. In this study, functional optical coherence tomography angiography (OCT-A) was used to dynamically assess the retinal microvasculature of X-linked IRD carriers.

**Methods:**

Genetically confirmed female carriers of IRDs (choroideremia or X-linked retinitis pigmentosa), and healthy women were recruited. Macular angiograms (3x3mm, Zeiss Plex Elite 9000) were obtained in 36 eyes of 15 X-linked IRD female carriers and 21 age-matched control women. Two tests were applied to test vascular reactivity: (i) mild hypoxia and (ii) handgrip test, to induce a vasodilatory or vasoconstrictive response, respectively. Changes to vessel density (VD) and vessel length density (VLD) were independently evaluated during each of the tests for both the superficial and deep capillary plexuses.

**Results:**

In the control group, the superficial and deep VD decreased during the handgrip test (p<0.001 and p=0.037, respectively). Mean superficial VLD also decreased during the handgrip test (p=0.025), while the deep plexus did not change significantly (p=0.108). During hypoxia, VD and VLD increased in the deep plexus (p=0.027 and p=0.052, respectively) but not in the superficial plexus. In carriers, the physiologic vascular responses seen in controls were not observed in either plexus during either test, with no difference in VD or VLD noted (all p>0.05).

**Conclusions:**

Functional OCT-A is a useful tool to assess dynamic retinal microvascular changes. Subclinical impairment of the physiological vascular responses seen in carriers of X-linked IRDs may serve as a valuable clinical biomarker.

## Introduction

**Figure 1:**
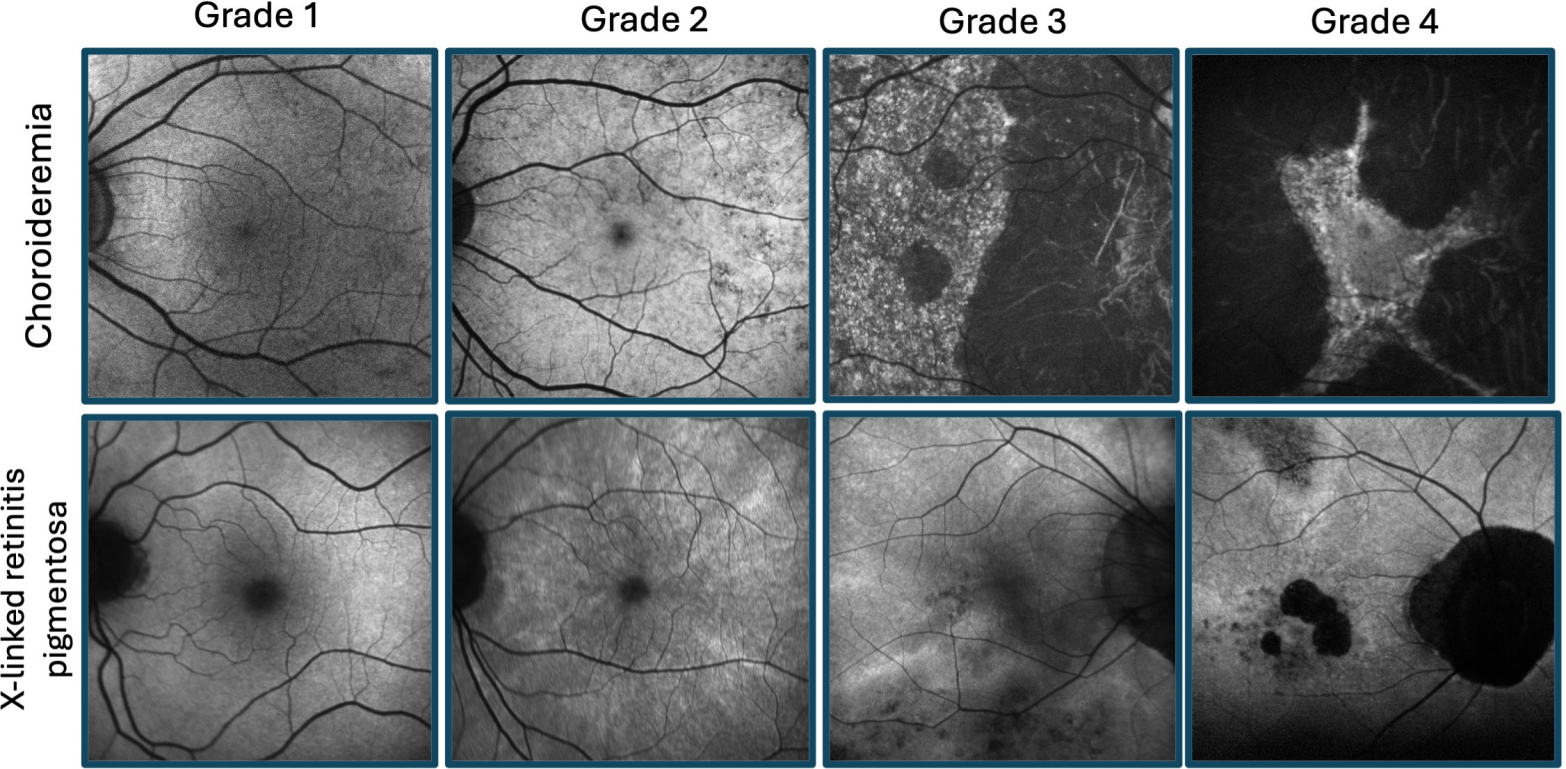
Retinal disease spectrum of female carriers of X-linked retinitis pigmentosa and choroideremia. Fundus autofluorescence imaging illustrating grades 1-4 for each condition, as previously described by Edwards et al (1) (fine, coarse, geographic, and male pattern phenotypes, respectively) and Nanda et al (2) (normal, radial, focal pigmentary retinopathy, and male pattern phenotypes, respectively).

Inherited retinal diseases (IRDs) are a group of heterogeneous conditions caused by pathogenic variants, typically leading to progressive vision loss. Males with X-linked IRDs, such as X-linked retinitis pigmentosa (XLRP) and choroideremia, experience severe retinal degeneration and eventual central vision loss at later-stages of disease. Female carriers of X-linked IRDs may present with a spectrum of retinal changes ranging from near normal retinae to severe retinal degeneration, the latter known as “male-pattern” degeneration (**Figure 1**) (1–3). This variability in retinal phenotype has been found to be attributed to X-chromosome inactivation (4), involving the random inactivation of one of two X-chromosomes in females (XX individuals) during early embryonic development (5).

One possible factor and/or consequence for the phenotypic heterogeneity seen in female carriers may be impaired vascular blood supply causing oxidative stress and subsequent production of reactive oxygen species, as seen in other degenerative conditions such as age-related macular degeneration (6, 7). Disruption to macular blood supply leads to hypoxia, retinal dysfunction, and disease (8). In support of this theory, markers of oxidative stress have previously been found in the aqueous humor of males with XLRP (9), plasma of males with choroideremia (10), and in the *chm^ru848^* zebrafish retina (11). However, further studies are required to establish clear causalities. Decreased perfusion density and vessel density in the superficial and deep capillary plexuses have been previously reported in people with IRDs, such as retinitis pigmentosa (12, 13), Stargardt disease (14, 15), and Best Vitelliform Macular Dystrophy (16), compared to healthy controls. Furthermore, people with choroideremia have also been found to have reduced deep capillary plexus vessel density, compared to healthy controls (17).

Although structural changes have been assessed in female carriers, impaired blood flow in the retinal vessels or oxidative stress have not been previously reported. There are currently limited studies reporting the integrity of the retinal microvasculature in female carriers of IRDs. A single study assessed retinal vascular structure in female choroideremia carriers and found no difference in the superficial and deep capillary plexuses, compared to healthy controls (18). Other studies have reported changes in choriocapillaris blood flow in female carriers of X-linked IRDs (19, 20). Altered physiological retinal vascular responses may serve as a biomarker of the risk of progression to sight-threatening disease.

Optical coherence tomography angiography (OCT-A) is an imaging modality used to provide non-invasive, high-resolution imaging of the retinal microvasculature. Motion contrast images are produced by the movement of red blood cells in the retinal blood vessels over sequential B-scans. OCT-A has been widely used to assess the retinal microvasculature in different retinal diseases (21), including diabetic retinopathy, macular degeneration, retinal vessel occlusion, schizophrenia and bipolar disorder (22–25). Recently, functional OCT-A has been used to dynamically assess retinal vascular reactivity in healthy individuals (26–30) and people with systemic disease (31–34). This technique involves inducing physiological changes to elicit vasoconstriction or vasodilation to assess the dynamic response of the retinal microvasculature. Detecting subtle microvascular alterations prior to symptoms manifestation could be advantageous for promptly diagnosing and treating various rare or systemic conditions (22, 35, 36). Hence, it could function as a versatile method employed by numerous healthcare professionals across different disciplines.

Functional OCT-A offers a distinct approach to visualizing and measuring tissue activity that surpasses other imaging techniques (37). This study aimed to evaluate the dynamic retinal microvascular responses in female carriers of X-linked IRDs with relatively mild retinal disease, using functional OCT-A.

## Methods

This prospective case-control study and informed consent process adhered to the tenets of the Declaration of Helsinki and received ethical approval from the University of Melbourne Human Research Ethics Committee (ID: 22809). Written informed consent was obtained from each participant prior to commencement of the study, which was undertaken at the Centre for Eye Research Australia, Victoria, Australia. Participants were recruited between November 2021 and January 2023.

### Study setting

Female carriers of X-linked IRDs (*RPGR*-associated XLRP and choroideremia), as confirmed by genetic testing, were referred by ophthalmologists, ocular genetics clinics, or responded to advertisements through the University of Melbourne and the Royal Victorian Eye and Ear Hospital. Advertisements for healthy age-matched female controls were distributed using the University of Melbourne staff newsletter, and other contacts of the authors.

Sample size was calculated considering a 5% clinically significant difference between the two cohorts in mean vessel density and 5% standard deviation, based on previous work by our team in individuals with diabetes (38). A minimum of 12 female carriers of X-linked IRDs and 12 healthy women were required for 80% power and an alpha value of 0.05.

### Inclusion and exclusion criteria

Participants were excluded if they had any of the following ocular conditions: severe retinal disease (i.e., male pattern phenotype), significant lens opacities (Lens Opacities Classification System III (39) equal to, or more than, grade 2), high refractive error (spherical equivalent above +4.00 or below -6.50 dioptres), glaucoma and/or ocular hypertension, neuro-ophthalmic disease, and previous intraocular surgery. Furthermore, participants were also excluded if they had any of the following systemic conditions: diabetes, uncontrolled hypertension (systolic >140 mmHg or diastolic >90 mmHg), inflammatory diseases, nephropathy, or other microvascular complications. Smokers (>20 cigarettes per day) and pregnant women were also excluded. Participants were also excluded if they were taking any vasoactive medication.

### Study design and interventions

Demographic data collected for all participants included age, smoking status, known diseases (systemic and ocular), current medication, history of intraocular surgery or trauma, symptoms of hypoxia during previous airplane flights. Researchers were unmasked to the carrier status of participants attending the study visits.

Eligible participants were asked to refrain from smoking, consuming caffeine and/or alcohol at least six hours prior to their appointment to reduce any vasoactive effects (40). Participants were randomly assigned into morning and afternoon appointments, to reduce the effect of any diurnal variations in systemic or ocular measurements.

All participants underwent an ophthalmic assessment comprised of best corrected visual acuity, slit lamp biomicroscopy, intraocular pressures (iCare TA01i tonometer, iCare Finland Oy, Vantaa, Finland), wide-field retinal fundus photography (Optos Daytona, Optos, Marlborough, MA, USA), and ocular biometry for axial length measurements (Zeiss IOLMaster 500, Carl Zeiss Meditec, Inc., Dublin, CA, USA). Body mass index and baseline measurements of blood pressure and oxygen saturation (SpO_2_) were also recorded.

**Figure 2:**
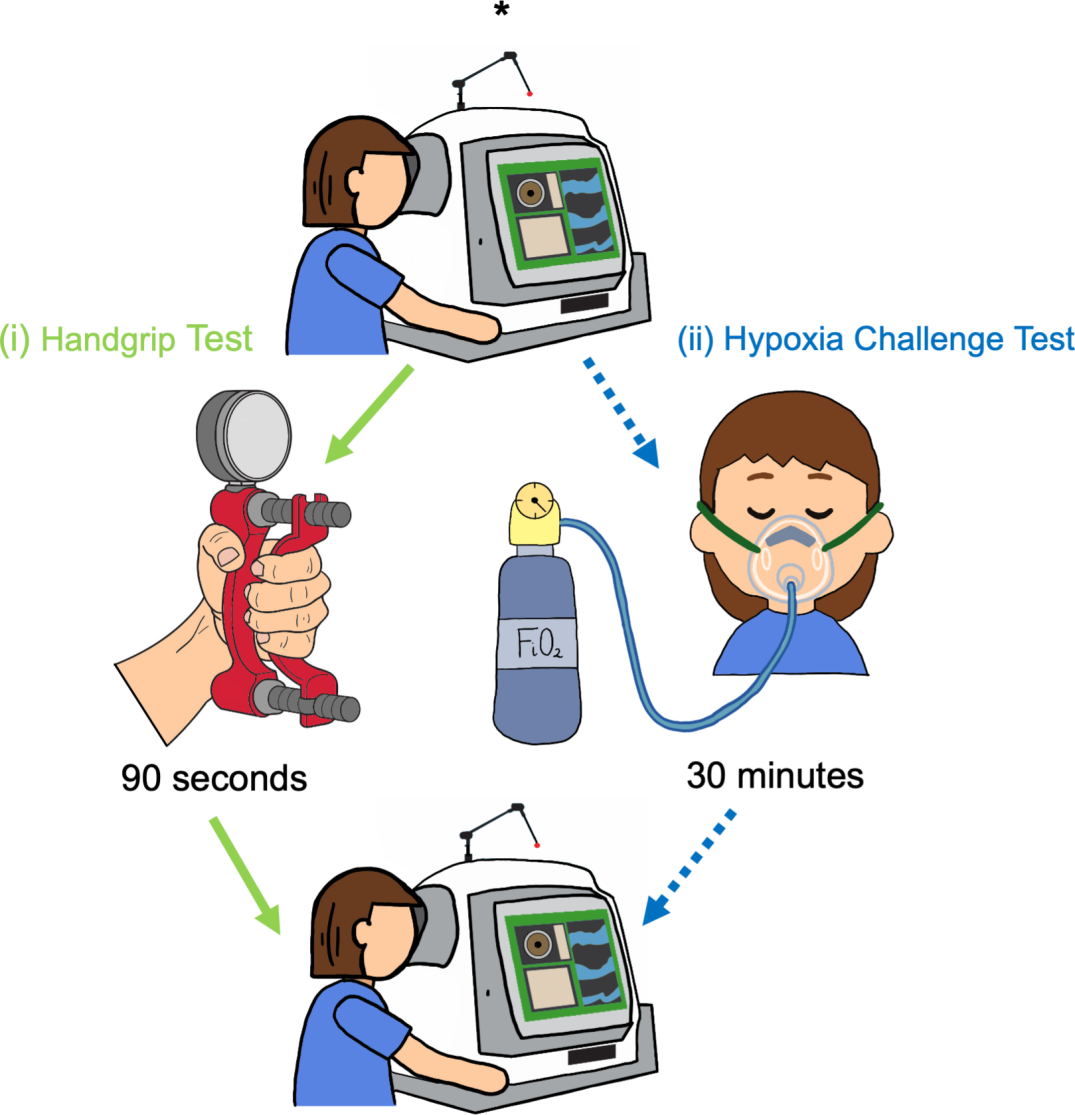
Study protocol. Baseline OCT-A images (*) were captured before each challenge test. Participants completed both challenge tests in the following order: (i) Handgrip test (green arrows) was performed first: OCT-A images were captured following 90 seconds of gripping the hydraulic dynamometer. A 10-minute break for recovery was provided after the handgrip test, and a second set of images were taken, to account for any residual variation in the vasculature following the rest period. (ii) Hypoxia challenge test (blue, dotted arrows): OCT-A images captured following 30 minutes of breathing fraction of inspired oxygen (FiO_2_).

The Zeiss Plex Elite 9000 (Carl Zeiss Meditec, Inc., Dublin, CA, USA) device was used to capture OCT-A images. The 3x3 mm angiography scans were captured at baseline and during the two dynamic test procedures (**Figure 2**). The built-in projection artifact removal software was used in order to increase the quality of the deep plexus angiograms. High quality (signal strength > 8/10 and no movement artefacts) images were captured at each time point for the right eye, followed by the left eye. Participants were provided with a 10-minute break between the two tests to ensure recovery from the handgrip test (41) before commencing the hypoxia challenge test.

### Handgrip test (isometric exercise) - vasoconstriction

The handgrip test protocol has been previously detailed (30). The test is a sympatheticomimetic stimulus leading to a steady increase in heart rate and blood pressure, causing a peripheral vasoconstrictive response.

Following detailed explanation of the study procedure, participants were placed in position on the OCT-A device with their elbows flexed at 90°, wrist and thumb facing upwards. Maximal grip force was measured prior to initiation of the handgrip test using a hydraulic dynamometer (Baseline 12-0241 LiTE, Baseline^®^, United States). Participants sustained grip at one third of their maximal grip force, for 3-5 minutes. OCT-A acquisition (3x3mm images) commenced after 90 seconds of continuous grip. A second clinician (CC, EC, or HK) measured blood pressure and SpO_2_ every minute during the test. As per the protocol, the test was ended if diastolic blood pressure was greater than 120mmHg, or any adverse events were noted (42).

### Hypoxia challenge test – vasodilation

The hypoxia challenge test was initially developed by the British Thoracic Society (43) and has been previously reported in detail (30). Participants breathed a gas mixture of 99.993% nitrogen through a 40% flow Venturi mask (Intersurgical EcoLite™, Intersurgical, Berkshire, United Kingdom) at 10L/min, corresponding to a fraction of inspired oxygen (FiO_2_) of 15%. Blood pressure was measured every 5 minutes from the initiation of hypoxic challenge, and SpO_2_ was continuously monitored. Following 30 minutes of hypoxic conditions, 3x3mm images were obtained for both eyes by a second clinician (SG). Testing was terminated if the diastolic blood pressure was greater than 120mmHg, or SpO_2_ was less than 90%.

### Image analysis

**Figure 3:**
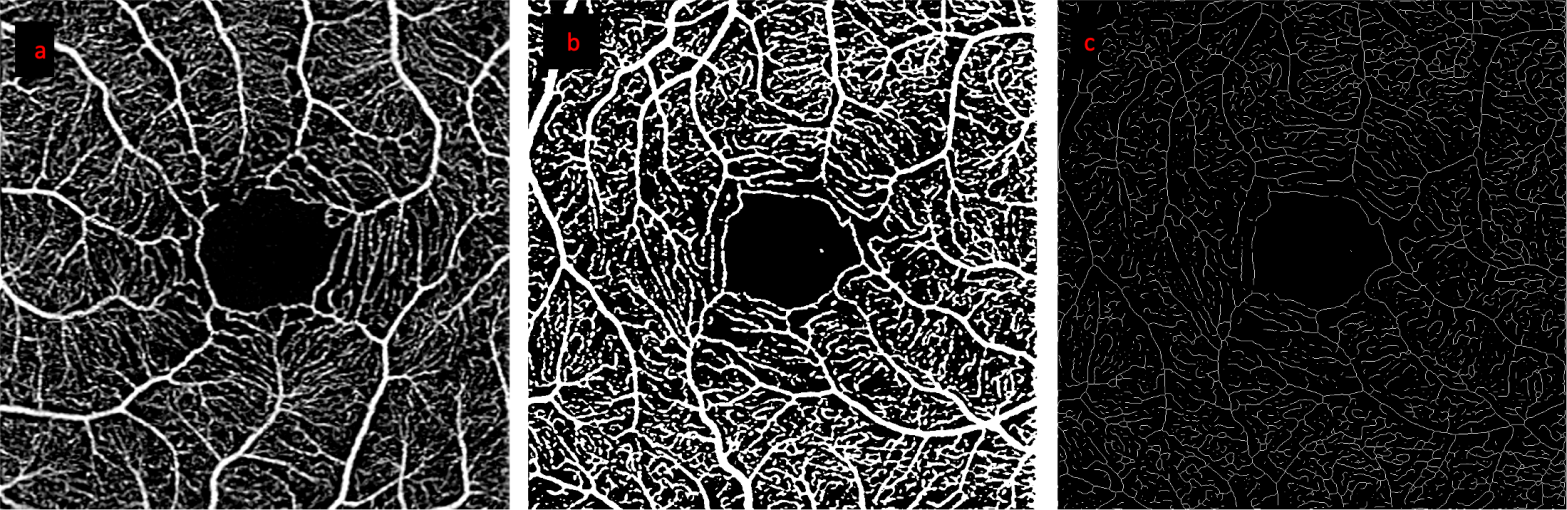
Step by step processing of OCT-A images. Images from a representative healthy control participant: **(A)** retinal angiogram of the superficial capillary plexus, **(B)** binarized image (used for VD calculation), **(C)** skeletonized image (used for VLD calculation).

Angiography images were exported for each test following projection artifact removal. Images were excluded if they had poor image quality (i.e., off-centre images, motion artefacts, or inaccurate segmentation). Single images were captured to represent each time point, due to the acquisition time of the macular scans (~45-60 seconds). As the last to be captured, the left eye was used to calculate perfusion density or otherwise known as vessel density (VD) and vessel length density (VLD) for both superficial and deep capillary plexuses, considering relatively longer exposure to the challenge test, compared to the right eye. Deep capillary plexus, as defined by the Zeiss Plex Elite, is a combination of the intermediate and deep capillary plexuses. Right eye images were not used to replace excluded left eye images. Images were analyzed using an automated feature of Fiji (ImageJ2, version 2.9.0) (44, 45) to binarize images (default, auto-threshold adjustment) and perform vessel calculations automatically, in order to avoid measurement bias associated with manual analysis (**Figure 3**). The Fiji software ‘measure’ function was used to calculate VD (ratio of white pixel by total number of pixel). The ‘skeletonize 2D/3D’ and ‘measure skeleton length tool’ plugins were used to skeletonize vessels and calculate VLD. VLD values were converted to mm^-1^, based on the total pixels and 3x3 mm area of the *enface* OCT-A images, as previously described (46–48).

### Statistical analysis

Statistical analyses were performed using GraphPad Prism version 9.5.1 (GraphPad Software, Boston, MA, USA). Normal distribution of values was tested using the Shapiro-Wilk test for normality. Differences in participant age, baseline data, and systemic measurements (i.e., blood pressure, heart rate, and SpO_2_) between female carriers and healthy controls were compared using an unpaired t-test or Mann-Whitney test, as appropriate. Repeated measures ANOVA was used to calculate changes in VD and VLD between baseline and challenge test results. An alpha value of 0.05 was used to determine statistical significance.

## Results

**Figure 4:**
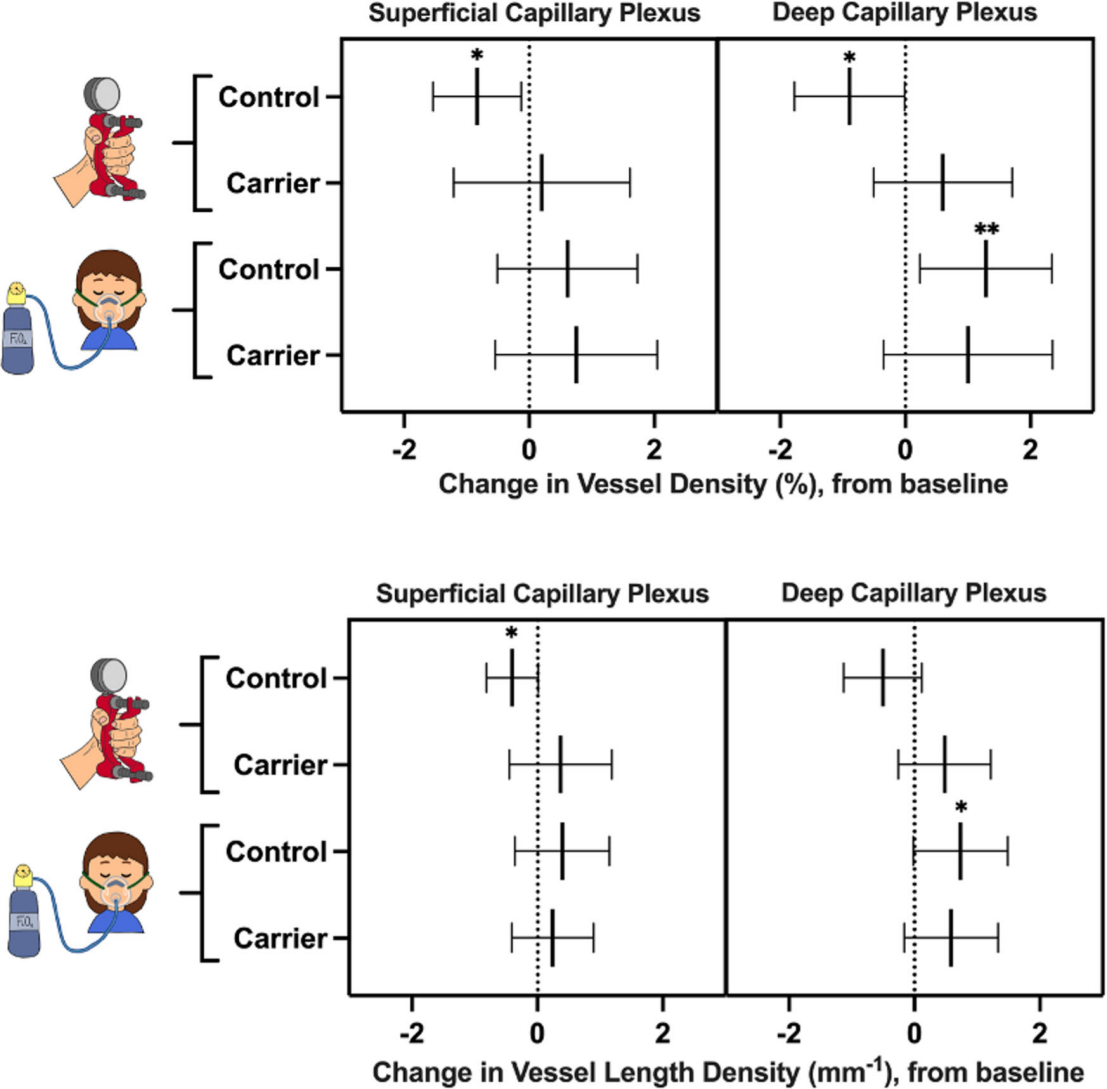
Change in vessel density (top) and vessel length density (bottom) of female carriers and healthy controls during the handgrip test and hypoxia challenge test, compared to baseline. Lines represent mean values and 95% confidence intervals. Asterisks represent statistically significant changes in vessel density and vessel length density after the challenge test, compared to baseline: * p<0.05; ** p<0.01.

**Figure 5:**
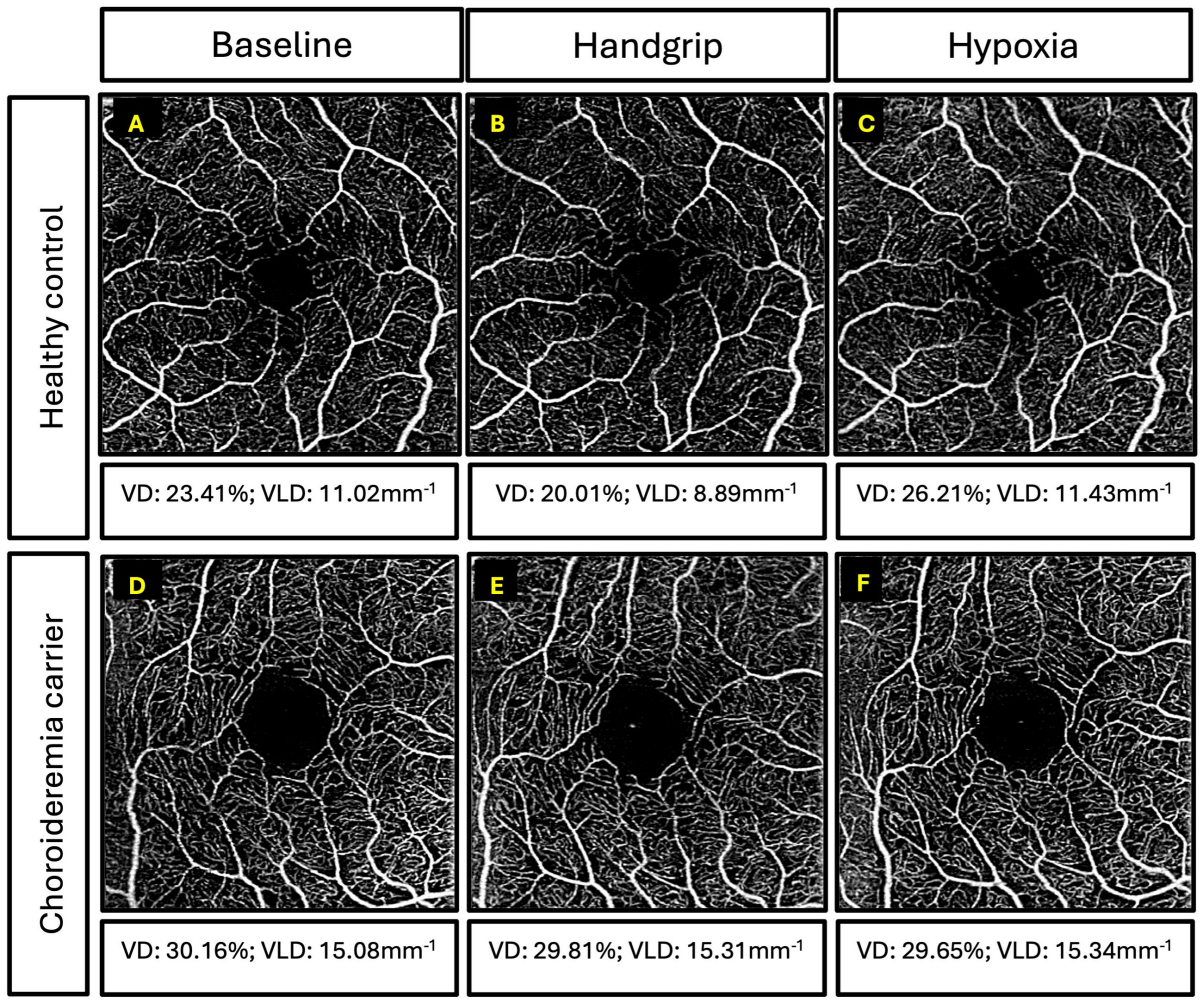
Examples of superficial capillary plexus images for participants. Top three panels are retinal angiography images of a 39-year-old healthy control and vessel density (VD) and vessel length density (VLD) measurements for: **(A)** baseline, **(B)** handgrip, and **(C)** hypoxia. Bottom three panels are retinal angiography images of a 55-year-old female carrier with coarse retinal phenotype and VD and VLD measurements for: **(D)** baseline, **(E)** handgrip, and **(F)** hypoxia.

Nineteen female carriers of X-linked IRDs with positive genetic test results were screened, however, 4 were excluded from participation (2 carriers had diabetes, 1 carrier with high myopia, and 1 due to vasoactive medication use). Thirty-six eyes from 15 genetically confirmed female carriers of X-linked IRDs (53% choroideremia and 47% *RPGR*-associated retinitis pigmentosa) and 21 healthy women were studied. Four angiography images from each participant group were excluded due to poor image quality. Baseline data and demographics are summarized in **Table 1**. There were no statistically significant differences in baseline parameters between groups for participant age, systolic arterial pressure (SAP), diastolic arterial pressure (DAP), heart rate (HR), body mass index (BMI), axial length, and intraocular pressure (IOP). Best corrected visual acuities were reduced in female carriers compared to healthy controls (median -0.02 logMAR vs -0.1 logMAR, p=0.001), although this difference was not clinically significant (carriers had 20/20 vision while healthy controls had vision one line better than 20/20). Changes in retinal vascular response compared to baseline in response to each challenge test are illustrated in **Figures 4**, **5** (for raw values, see **Supplementary Table 1**). At baseline, VD and VLD were not significantly different between healthy controls and female carriers for superficial and deep capillary plexuses (p>0.05) (**Table 1**).

**Table 1:** Demographics and baseline data for healthy women and female carriers of X-linked IRDs.

Parameter (mean ± SD)	Controls (n=21)	Female carriers (n=15)	P value
Age, years^a^	45 (30 – 54)	48 (32.5 – 55)	0.99
SAP, mmHg	115 ± 12	118 ± 14	0.62
DAP, mmHg	80 ± 11	80 ± 8	0.96
HR, bpm	72 ± 12	73 ± 11	0.86
BMI	25.05 ± 5.29	26.87 ± 6.16	0.36
Axial length, mm	23.81 ± 1.10	23.24 ± 0.92	0.12
Visual acuity, logMAR^a^	**-0.10 (-0.16 – -0.06)**	**-0.02 (-0.08 – 0.13)**	**0.001**
IOP, mmHg	15.26 ± 2.49	15.01 ± 2.94	0.78
Baseline superficial capillary plexus			
- Vessel density, % ^-^ Vessel length density, mm^-1^	24.56 ± 3.10 11.89 ± 1.79	25.87 ± 3.75 12.75 ± 2.16	0.27 0.21
Baseline deep capillary plexus			
- Vessel density, % ^-^ Vessel length density, mm^-1^	30.91 ± 2.90 16.11 ± 1.70	28.84 ± 3.84 14.89 ± 2.22	0.08 0.08
Retinal severity grading^b^	–	Grade 1: 5 (33%) Grade 2: 8 (53%) Grade 3: 2 (13%) Grade 4: 0 (0%)	–
Refractive error	OD: -1.25 ± 2.50 OS: -1.00 ± 2.50	OD: -0.25 ± 2.50 OS: -0.25 ± 2.75	0.14 0.14
OCT-A signal strength^c^	**8.3 ± 0.7**	**8.8 ± 0.6**	**0.02**

a Values represented as median (interquartile range).

b Severity scale score of 1-4 used to capture retinal phenotypic spectrum from near normal retinae to severe retinal degeneration based on classification introduced by Nanda et al (2) for RPGR-associated RP and Edwards et al (1) for CHM.

c OCT-A signal for left eye only, as these images were used in the analysis.

Statistically significant values are in bold.

Unpaired t-test and Mann-Whitney test were used to compare the two groups for normally and non-normally distributed parameters, respectively. Visual acuity was not clinically significant (carriers had 20/20 vision while healthy controls had vision one line better than 20/20).

BMI, body mass index; DAP, diastolic arterial pressure; HR, heart rate; IOP, intraocular pressure; OCT-A, optical coherence tomography angiography; SAP, systolic arterial pressure, SD, standard deviation.

### Retinal microvascular response

#### Handgrip test

In healthy controls, superficial and deep capillary plexuses demonstrated significant decrease in VD in response to the handgrip test (p-values of 0.01 and 0.02, respectively), compared to baseline (**Figure 4**). The superficial capillary plexus VLD decreased during the handgrip test compared to baseline (p=0.03). Although there was a trend towards a reduction in VLD in the deep capillary plexus, this did not achieve statistical significance (p=0.05). The female carrier cohort did not show the expected vasoconstrictive response (VD or VLD) to handgrip in either the deep capillary plexus or superficial capillary plexus (p>0.05).

#### Hypoxia challenge test

In the healthy cohort, the deep capillary plexus significantly increased in VD and VLD during the hypoxia challenge test (p-values of 0.01 and 0.03, respectively), despite no statistically significant changes in the superficial capillary plexus, compared to baseline (p>0.05) (**Figure 4**). Female carriers of X-linked IRDs did not show any statistically significant physiological retinal vascular changes in response to hypoxic conditions (p>0.05) (**Figure 4**).

### Systemic response

The expected physiologic systemic response was observed during the handgrip test in both groups, with increases in SAP, DAP, and HR, compared to baseline, which were not significantly different between controls and female carriers. The expected decrease in SpO_2_ was observed during the hypoxia challenge test in both groups, however, there were no significant changes in SAP, DAP, and HR, compared to baseline. Furthermore, these observed changes were not significantly different between controls and female carriers.

### Sub-analysis of near normal retinal phenotype

Dynamic retinal microvascular response of female carriers with the mildest phenotype, near normal retinae (n=5), were compared with that of healthy women. In response to the handgrip and hypoxia challenge tests, only a single carrier showed the expected change in VD and VLD of the superficial and deep capillary plexuses, as seen in healthy women. Whereas the remaining 4 had no significant changes in vessel density.

## Discussion

This study aimed to assess dynamic retinal microvascular changes in female carriers of X-linked IRDs with relatively mild retinal disease, using a functional OCT-A protocol. Changes in physiological conditions induced by isometric exercise and hypoxia elicited significant changes in VD and VLD in healthy women, compared to baseline. However, such dynamic retinal changes were not seen in female carriers of X-linked IRDs, despite equivalent changes in systemic measurements for SAP, DAP, HR, and SpO_2_ in both participant groups. There were no significant differences between VD and VLD between female carriers and healthy controls at baseline. Accordingly, carrier status is associated with dysregulation of the retinal vasculature as opposed to structural vascular impairment. It follows that altered retinal vascular responses may serve as markers of carrier status and potentially guide in understanding the underlying vascular impairment in retinal disease.

Control participants in the current study demonstrated the expected retinal vascular responses to both challenge tests, as reported by Sousa et al. (33) On the contrary, Brinkmann et al. reported no significant changes in retinal perfusion in the superficial and deep capillary plexuses in healthy individuals following isometric exercise (49). These differences may be attributed to differences in OCT-A imaging devices or image analysis methods. The study by Sousa et al. used RTVue XR Avanti OCT-A and the study by Brinkmann et al. used the Spectralis OCT Angiography Module which both have different wavelength, acquisition speed, and axial resolution to the Zeiss Plex Elite, used in the current study (50). A study by Arya et al. compared VD and vessel length density (i.e., VLD) between three OCT-A devices (RTVue XR Avanti, Cirrus HD-OCT 5000, and Plex Elite 9000) and found significant differences in OCT-A metrics (50). Therefore, comparison of vessel density between different devices is not ideal due to differences in accuracy, repeatability and reproducibility (50). The Zeiss Plex Elite 9000 has an axial (optical) and transverse resolution of 6.3µm and 20µm, respectively, compared to the RTVue Avanti OCTA device which has an axial and transverse resolution of 5µm and 15µm, respectively. These differences in device specifications may influence overall image quality, and certainly limit the ability to detect a change in vessel diameter of small retinal capillaries. However, the aim of functional OCT-A is to alter the threshold for detection of blood flow within the retinal microvascular network, so that the blood flow of a retinal capillary close to this threshold is more of less likely to be detected by the device with each of the stimulus and therefore appear or not in the angiogram. The total average change in the perfusion metrics will reflect this. Furthermore, the RTVue XR Avanti device provides automated calculations of VD, rather than relying on manual calculations using third-party software (i.e., ImageJ). Currently, it is difficult to postulate which device and analysis method is superior, as there is a need to determine which OCT-A device and analysis method accurately represents true vascular metrics.

There are many methods available to elicit a dynamic response in the retinal microvasculature. The handgrip test and hyperoxic conditions have been previously used to induce retinal vasoconstriction (27, 30, 51, 52), while the flicker test and hypoxia have been reported to illicit a vasodilatory response (27, 30, 53). Currently, these techniques are limited to research use, however they may have clinical applicability with standardization of methods and reporting of test findings. The hypoxic challenge and handgrip tests were selected for the present study as they are fast, inexpensive, reproducible, and easily deployed in most clinical settings. The hypoxia challenge test is standardized by the British Thoracic Society to assess whether individuals with stable respiratory disease are suitable for a long-haul flight by exposing them to reduced oxygen levels similar to those encountered in an airplane cabin (54). The handgrip test is used to assess cardiovascular autonomic function (42), and has been used for a wide variety of clinical indications (33, 51, 52). Despite their convenience, both tests have their limitations. It may be challenging for some individuals to sustain the required handgrip for the duration of the test. The relatively longer duration of the hypoxia challenge test can limit its use in some contexts and the Venturi mask may restrict positioning of the participant at the OCT-A device. These limitations were not problematic in the current study, and we did not have any adverse events using either technique.

The two groups in the current study were well matched for age, gender, BMI, systemic cardiovascular measures, axial length, and IOP. Best-corrected visual acuity was slightly lower in controls than in carriers, however this difference was not clinically significant (logMAR of -0.10 in controls and -0.02 in female carriers, p=0.001). Most carriers in the current study had grade 1 (33%) or 2 (53%) retinal disease severity, which are mild retinal phenotypes. Although the results of the current study may not be applicable to all retinal phenotypes seen in carriers, they suggest that vascular dysregulation is manifest in carriers with mild retinal disease, potentially indicating that this may be a manifestation of carrier status. Longitudinal studies are needed to establish a causal relationship between vascular dysregulation and retinal phenotype severity. A sub-analysis of the mildest, grade 1 female carriers (n=5) showed that only a single carrier had the expected physiological response to the challenge tests, while the remaining four carriers did not. Statistical analyses were not performed due to the limited sample size, however, this further suggests that vascular reactivity may be impaired in the presence of the disease-causing genetic variant, despite the degree of retinal disease severity. Future studies should consider a larger sample size to determine whether retinal disease severity impacts vascular reactivity. Conducting this study protocol on female carriers with severe retinal phenotypes is likely to be precluded by impaired fixation due to visual impairment and the confounding effects of marked structural alterations seen in individuals with severe retinal degeneration. Therefore, female carriers with male-pattern degeneration were not included in the current study, but it may be of interest in future studies if the image acquisition challenges can be addressed.

## Limitations

The small number of female carriers of X-linked IRDs in the current study is attributable to several factors: (i) *RPGR-*associated XLRP and choroideremia are rare conditions; (ii) female carriers do not undergo routine genetic testing, and genetic confirmation of carrier status was required for participation in the study; and (iii) strict inclusion and exclusion criteria further limiting the number of eligible carriers. The genetic cause and underlying cellular dysfunction differ between *RPGR-*associated XLRP and choroideremia. However, since female carriers of both conditions present a spectrum of retinal disease severity, the current study was interested in the effect of carrying the disease-causing gene on vascular reactivity without obvious severe retinal degeneration (i.e., male pattern phenotype). Future studies with additional carriers of both conditions are required to compare each condition against healthy controls. Precisely why some carriers exhibit a more severe retinal phenotype is poorly understood. The current findings suggest that aberrant retinal vascular regulation may be biomarker for female carrier status. Longitudinal assessment of carriers from a young age may clarify whether the degree of vascular dysregulation may predict female carrier phenotypic severity.

As an inherent limitation of OCT-A, VD and VLD are not true representations of blood flow (55, 56). However, several studies have previously used such parameters to represent vasodilation and vasoconstriction (26, 27, 29, 32, 51, 57, 58). There are currently several limitations to reporting of OCT-A findings due to: (i) motion artifacts, projection artifact removal and segmentation errors seen in eyes with different retinal diseases using current OCT-A devices (59); and (ii) the absence of a standardized system for image acquisition and analysis. As discussed, different OCT-A device platforms have different performance metrics and operational parameters which can make inter-device comparisons challenging. Furthermore, there are differences in the quantitative results obtained for a given image set using different software packages such as the Zeiss ARI Network (60), ImageJ, MATLAB, or automated values obtained from select imaging devices. Different methods were compared and automated ImageJ analysis was found to be the most reliable and repeatable. A study by Ishii et al. found that foveal avascular zone calculations measured by an automated software in ImageJ is comparable to manual measurements, and significantly different to the values measured using tools available on the Zeiss ARI Network (61). Therefore, we recognize the need for a standardized method for analyzing and reporting OCT-A findings as well as validated methods to enable comparisons between devices. Prior studies have commonly employed the practice of averaging multiple OCT-A images to enhance the precision of quantifying vascular measures (38, 62). However, in the current study, achieving this was not feasible, as the retinal response triggered by the 90-second handgrip test would dissipate within the time required for acquiring multiple images (approximately 45-60 seconds per image). For functional OCT-A to be applied in clinics, the technology should be optimized to reliably provide repeated measures of VD and VLD within the same subject and in maculae with retinal disease. We suggest that in addition to the eye tracking feature enabling follow up imaging, matching greyscale between angiography scans within the same subject is an important feature for quantification of OCTA metric between repeated measures.

## Conclusion

This study reiterates the value of functional OCT-A for detecting changes in retinal vascular regulation in the absence of retinal vascular network abnormalities. Our findings suggest female carriers of X-linked IRDs exhibit subclinical impairment of physiological retinal vascular responses, which may indicate retinal microvasculature sensitivity to presence of the disease-causing variant on the X chromosome. Although the causal relationship between vascular changes and genetics remains unclear, our study suggests these dynamic vascular changes may serve as a biomarker for disease severity and prognosis in female carriers.

## Data availability statement

The original contributions presented in the study are included in the article/**Supplementary Material**. Further inquiries can be directed to the corresponding author.

## Ethics statement

The studies involving humans were approved by University of Melbourne Human Research Ethics Committee. The studies were conducted in accordance with the local legislation and institutional requirements. The participants provided their written informed consent to participate in this study. Written informed consent was obtained from the individual(s) for the publication of any potentially identifiable images or data included in this article.

## Author contributions

SG: Conceptualization, Data curation, Formal analysis, Investigation, Methodology, Project administration, Software, Supervision, Validation, Visualization, Writing – original draft, Writing – review & editing. XH: Formal analysis, Investigation, Visualization, Writing – review & editing. CC: Methodology, Writing – review & editing. EC: Methodology, Writing – review & editing. HK: Methodology, Writing – review & editing. JJ: Supervision, Writing – review & editing. PV: Supervision, Writing – review & editing. TL: Supervision, Writing – review & editing. LA: Conceptualization, Funding acquisition, Resources, Supervision, Writing – review & editing. DS: Conceptualization, Funding acquisition, Investigation, Resources, Software, Supervision, Validation, Writing – review & editing.
