# Supplementary material for: Retinal vascular reactivity in carriers of X-linked inherited retinal disease – a study using optical coherence tomography angiography
Source: Front Ophthalmol (Lausanne). 2024 Jul 9;4:1415393. doi: 10.3389/fopht.2024.1415393 (PMC11263797; doi:10.3389/fopht.2024.1415393)
Supplement: Supplementary file 1 [file Table_1.docx]

**Supplementary Materials**

| **median (IQR)** | **Female carriers (n=15)** | **Healthy controls (n=23)** | **P-value** |
| --- | --- | --- | --- |
| **Age** | 48 (33.25 – 55.75) | 45 (30-54) | 0.99 |
| **Vessel Density (VD)** | | |  |
| **SCP_Baseline1** | 26.52 (24.17 – 29.05) | 23.69 (21.92 – 26.74) | 0.27 |
| **SCP_Handgrip** | 24.03 (22.41 – 29.67) | 24.45 (21.01 – 26.8) |  |
| *p*-value | 0.36 | **0.01** |  |
| **SCP_Baseline2** | 26.52 (22.96 – 29.04) | 23.64 (22.08 – 26.29) |  |
| **SCP_Hypoxia** | 26.75 (25.92 – 27.76) | 26.33 (22.77 – 27.42) |  |
| *p*-value | 0.18 | 0.13 |  |
| **DCP_Baseline1** | 2896 (2811 – 30.88) | 31.5 (27.9 – 33.43) | 0.08 |
| **DCP_Handgrip** | 29.13 (28.13 – 30.76) | 30.72 (28.33 – 31.99) |  |
| *p*-value | 0.24 | **0.02** |  |
| **DCP_Baseline2** | 28.28 (27.74 – 29.69) | 30.56 (28.39 – 31.85) |  |
| **DCP_Hypoxia** | 30.04 (27.31 – 32.78) | 32.06 (29.57 – 34.93) |  |
| *p*-value | 0.12 | **0.01** |  |
| **Vessel Length Density (VLD)** | | |  |
| **SCP_Baseline1** | 13.05 (11.49 – 14.28) | 11.73 (10.55 – 13.07) | 0.21 |
| **SCP_Handgrip** | 11.95 (11.16 – 15.08) | 11.56 (10.03 – 13.31) |  |
| *p*-value | 0.09 | **0.03** |  |
| **SCP_Baseline2** | 13.05 (11.05 – 14.15) | 11.56 (10.29 – 12.41) |  |
| **SCP_Hypoxia** | 12.92 (12.27 – 14.41) | 12.43 (10.75 – 13.94) |  |
| *p*-value | 0.36 | 0.14 |  |
| **DCP_Baseline1** | 14.97 (14.45 – 15.56) | 16.54 (14.53 – 17.42) | 0.08 |
| **DCP_Handgrip** | 15.22 (14.18 – 16.43) | 15.87 (14.31 – 16.89) |  |
| *p*-value | 0.11 | 0.05 |  |
| **DCP_Baseline2** | 14.79 (14.09 – 15.48) | 16.13 (14.49 – 16.9) |  |
| **DCP_Hypoxia** | 16.4 (14.76 – 17.15) | 16.69 (15.02 – 18.92) |  |
| *p*-value | 0.10 | **0.03** |  |
